# Gene Expression Profile Analysis of the Molecular Mechanism of HOXD10 Regulation of Epithelial Ovarian Cancer Cells

**DOI:** 10.7150/jca.90970

**Published:** 2024-01-01

**Authors:** Chia-Yi Hsu, Ching-Chou Tsai, Hsiao-Yun Lin, Hsiang-Ling Chen, Yu-Che Ou, Ping-Hsuan Chiang, Jau-Ling Suen, Eing-Mei Tsai

**Affiliations:** 1 Department of Obstetrics and Gynecology, Kaohsiung Medical University Hospital, Kaohsiung Medical University, Kaohsiung City 807, Taiwan.; 2 Graduate Institute of Medicine, College of Medicine, Kaohsiung Medical University, Kaohsiung City 807, Taiwan.; 3 Department of Obstetrics and Gynecology, Kaohsiung Chang Gung Memorial Hospital and Chang Gung University College of Medicine, Kaohsiung 833, Taiwan.; 4 Graduate Institute of Clinical Medicine, College of Medicine, Kaohsiung Medical University, Kaohsiung City 807, Taiwan.; 5 Department of Obstetrics and Gynecology, Chia-Yi Chang Gung Memorial Hospital, Chiayi 613, Taiwan.; 6 Department of Internal Education, Kaohsiung Chang Gung Memorial Hospital, Kaohsiung 833, Taiwan.

**Keywords:** epithelial ovarian cancer, HOXD10, microarray, oxidative phosphorylation

## Abstract

Epithelial ovarian cancer (EOC) is the most common type of ovarian cancer. Although studies have reported that downregulation of HOXD10 expression may contribute to the migration and invasion abilities in EOC, much about its regulation remains to be fully elucidated. The present study aimed to identify different gene expression profiles associated with HOXD10 overexpression in EOC cells. The present study confirmed that HOXD10 overexpression effectively inhibited the proliferation and motility of the TOV21G and TOV112D cells. Further, we overexpress HOXD10 in TOV112D cells, the different gene expression (DEGs) profiles induce by HOXD10 was analyze by the Human OneArray microarray. Gene Ontology (GO) and Kyoto Encyclopedia of Genes and Genomes (KEGG), ingenuity pathway analysis (IPA) was used to perform the pathway enrichment analysis for the DEGs. Integrated bioinformatics analysis showed that the DEGs were enriched for terms related to oxidative phosphorylation and mitochondrial function pathways. Dysfunction oxidative phosphorylation metabolic pathway occurs frequently in many tumors. We validated the expression of* NDUFA7*, *UQCRB* and *CCL2* using qPCR, involving in metabolism-related pathway, were significantly changed by HOXD10 overexpression in EOC. The detailed regulatory mechanism that links HOXD10 and the oxidative phosphorylation genes is not yet fully understood, our findings provide novel insight into HOXD10-mediated pathways and their effects on cancer metabolism, carcinogenesis, and the progression of EOC. Thus, the data suggest that strategies to interfere with metabolism-related pathways associated with cancer drug resistance could be considered for the treatment of ovarian tumors.

## Introduction

Epithelial ovarian cancer (EOC) is one of the most common gynecological cancers, and it has a high mortality rate. Endometriosis has been reported to undergo malignant transformation and is associated with and increased risk of EOC, mainly endometrioid and clear cell subtypes 1, 2. At present, two major clinical challenges result in a high mortality rate for women with ovarian cancer. One is a lack of specific symptoms, making it difficult to identify and treat EOC at an early stage. Thus, the majority of patients have with late-stage EOC by the time of diagnosis. The other is that, despite there being many chemotherapeutic agents and treatment protocols available, the overall long-term survival of women with EOC remains poor 3, 4. Therefore, it is imperative to explore new avenues for the early diagnosis and targeted treatment of ovarian cancer to improve the disease outcome.

HOXD10 is a member of the homeobox (*HOX*) gene family, the members which encode transcription factors with HOX DNA-binding regions. HOXD10 is abnormally expressed in many malignant tumors, including breast, bladder, ovarian, and colon cancers 5-7. Several studies have reported that HOXD10 is related to migration, invasion, and proliferation in tumor cells 6, 7. Furthermore, HOXD10 can directly affect extracellular matrix remodeling and cell migration during angiogenesis through suppress MMP14 and uPAR 8. Our previous study showed that the low expression of miR-381 in endometriosis-associated clear cell and endometrioid ovarian cancer could be reversed upon increased HOXD10 expression, which drives the expression of miR‑381, which in turn may affect the proliferation and metastasis of ovarian cancer cells 9. Therefore, in the present study, a comprehensive analysis was performed to gain insight into the potential underlying genetic alternations related to HOXD10 modification in ovarian cancer using gene expression array and bioinformatics analysis. HOXD10 was involved in modulating metabolism-related pathways, such as oxidative phosphorylation, mitochondrial function, and the granzyme A signaling pathway. Growing evidence indicates that the dysregulated metabolism of cancer cells is a likely cause of chemotherapeutic resistance 10. These data provide insights into new strategies to interfere with metabolism-related pathways for the treatment of ovarian tumors.

## Materials and methods

### Cell culture

Human ovarian cancer cell lines, TOV21G, TOV112D, JHOC5 and OVK18 obtained from the American Type Culture Collection (Rockville, MD, USA). TOV21G and TOV112D cells were grown in complete Medium 199 (Gibco-Life Technologies, Carlsbad, CA, USA) and MCDB 105 medium (Sigma-Aldrich, St. Louis, MO, USA) (1:1, v/v) supplemented with 15% FBS (Gibco-Life Technologies) and 1% penicillin/streptomycin (Gibco-Life Technologies). JHOC5 were culture in in Dulbecco's Modified Eagle's Medium: Nutrient Mixture F12 (Gibco-Life Technologies) with 10% FBS (Gibco-Life Technologies) and 1% penicillin/streptomycin (Gibco-Life Technologies). OVK18 cells were cultured in Minimum Essential Medium (Gibco-Life Technologies) with 10% FBS and 1% penicillin/streptomycin. Immortalized normal human ovarian surface epithelial cells (IOSE) were a generous gift from Dr. Chan (National Chung Cheng University, Chiayi, Taiwan) and were grown in complete Medium 199 and MCDB 105 medium (1:1, v/v) supplemented with 10% FBS, 10 ng/mL EGF, 400 ng/mL hydrocortisone (Sigma-Aldrich), and 1% penicillin/streptomycin. All cells were cultured in a humidified incubator at 37°C with 5% CO2.

### Plasmids and transfection

Cell lines were growth to ~50% confluency. They were then transfected with a scramble control shRNA (cat. no. TRCN 0000274091, National RNAi Core Facility at the Institute of Molecular Biology, Academia Sinica, Taipei), shRNA against HOXD10 (cat. nos. TRCN 0000274091TRCN0000274093, and TRCN 0000274028; National RNAi Core Facility at the Institute of Molecular Biology, Academia Sinica, Taipei), control plasmid (pCDNA3) or HOXD10-pCDNA plasmid (gift from Corey Largman, Addgene plasmid # 21007) using Turbofect Transfection Reagent (Thermo Fisher Scientific, Inc., Waltham, MA, USA). After 24h, the medium was changed to fresh growth medium. After another 24 h, all cells achieved about 70-80% transfection efficiency before being subjected to the assays.

### RNA extraction, cDNA synthesis, and qPCR

Total RNA was extracted from cells using TRIzol (Invitrogen; Thermo Fisher Scientific, Inc.) according to the manufacturer's protocol, and cDNA was synthesized using the HiSenScript RH (-) RT PreMix Kit (cat. 25087; iNtRON Biotechnology, Seongnam, Korea). Quantitative real-time PCR (qPCR) analysis was carried out using the ORA™ SEE qPCR Green ROX L Mix (2_X_, SYBR-Green, highQu GmbH, Kraichtal, Germany), using the Applied Biosystems 7500 Real-Time PCR System (Thermo Fisher Scientific, Inc.). Quantification and normalization of the amplified products were performed using *18S* as a control. The primers were synthesized by Genomics BioSci and Tech Ltd (New Taipei City, Taiwan): *18S* forward, 5'-CATGGCCGTTCTTAGTTGGT-3' and reverse', 5'-CGCTGAGCCAGTCAGTGTAG-3'; *HOXD10* forward, 5'-ATAAGCGCAACAAACTCATTTCG-3' and reverse, 5'-ATATCGAGGGACGGGAACCT-3'; *NDUFA7* forward, 5'-GAACTCAGCCTCCTCCCAAG-3' and reverse, 5'-AGTCACCGCCTTCTTCTCAG-3'; *UQCRB* forward, 5'-TAAGAGGGCACTGGACCTGAAC-3' and reverse, 5'-CATGATTACTTCTTTGCCCATTCTT-3'; *CCL2* forward, 5'-GCAGCAAGTGTCCCAAAGAAG-3' and reverse, 5'-TCTTCGGAGTTTGGGTTTGCT-3'. Thermocycling conditions for gene expression were as follows: 40 cycles of denaturation at 95°C for 15 s, annealing at 60°C for 60 s, extension at 72°C for 30s, and a final extension at 72°C for 5 min. In addition, *18S* was used as the endogenous control. The 2^-ΔΔCq^ method was used for the calculation of relative expression 11.

### Cell viability analysis

Cell Counting Kit-8 (CCK-8; Fluka, Honeywell, Charlotte, NC, USA.) was used to study the viability of the ovarian cancer cell lines. Cells were seeded in 96-well plates at a density of 5 × 10^3^ cells per well, with a total volume of 100 μl of medium with 15% (v/v) FBS and were incubated for transfected with plasmids, shRNA, or controls as described above. The following day, 10 μl of CCK‑8 reagent was added to each well and maintained for a further 2 h at 37°C. Finally, cells were assayed with an ELISA reader at 450 nm (reference, 650 nm) (Multiskan EX; Thermo Fisher Scientific, Inc.). Three independent experiments were conducted in each case.

### Migration assays

For migration assays, 1 × 10^4^ ovarian cancer cells were suspended in serum-free culture medium and seeded in an upper chamber of a Transwell insert with and 8-µm pore size (no. 353097, BD Biosciences, Franklin Lakes, NJ, USA), and 700 μL culture medium with 15% FBS was added to the lower compartment. Following incubation at 37°C for 18 h, cells that had passed through a were fixed with 4% paraformaldehyde for 10 min, and cells in the top chamber were removed by wiping with cotton swabs. Invading cells were stained for 20 min with 0.1% crystal violet. The of migrating cells were quantified by counting five randomly selected image fields under a phase‑contrast microscope. In each case, three independent experiments were performed.

### Western blot analysis

Proteins were harvested from ovarian cell lines using a radio-immunoprecipitation assay (RIPA) buffer (EMD Millipore, Billerica, MA, USA). Protease and phosphatase inhibitors were added to the RIPA buffer (Roche Applied Science, Madison, WI, USA). Protein quantification was performed using the Pierce BCA Protein Assay Kit (Thermo Scientific, Rockford, IL, USA). Then, for each sample, 30 μg of protein was separated by 10% SDS-PAGE at 100 V and transferred onto nitrocellulose membranes at 90 V for 1.5 h. Membranes were blocked at room temperature for 1 h with 5% nonfat milk in phosphate-buffered saline plus 1% Tween 20 (PBST). After three washes for 10 min each with PBST, membranes were incubated overnight at 4℃ with anti-HOXD10 (1:500, cat. no. TA800777; OriGene Technologies, Inc., Rockville, MD, USA), or anti-actin (1:5,000, cat. no. A5316; Sigma‑Aldrich). The membranes were subsequently incubated for 1 h at room temperature with horseradish peroxidase conjugated goat anti-rabbit or anti-mouse secondary antibodies (Santa Cruz Biotechnology, Inc., Dallas, TX, USA). Enhanced chemiluminescence reagents (EMD Millipore) were used for immunodetection.

### Human oligonucleotide DNA microarray analysis

The Human Whole Genome OneArray v7 (Phalanx Biotech Group, Zhubei city, Taiwan) contains 29,204 DNA oligonucleotide probes, with each probe designed as a 60-mer in the sense orientation. Among the probes, 28,264 correspond to the annotated genes in the RefSeq v70 and Ensembl v80 databases, and 940 control probes are also included. A detailed description of the gene array list is available from http://www.phalanx.com.tw/Products/HOA_Probe.php.

### Microarray analysis

Briefly, total RNA was extracted from HOXD10-transfected TOV112D cells as the experimental group (HD1, HD2) and from mock-transfected TOV112D cells (ND1, ND2) as the control group using TRIzol according to the manufacturer's protocol. Fluorescent antisense RNA (aRNA) targets were prepared from 1 μg of total RNA. Second-strand cDNA synthesis and cDNA cleanup were carried out according to the OneArray Amino Allyl aRNA Amplification Kit (Phalanx Biotech Group). The entire purified cDNA was used in the *in vitro* transcription reaction. Fluorescent targets with Cy5 dye (GE Healthcare) were hybridized to the Human Whole Genome OneArray with Phalanx hybridization buffer using the Phalanx Hybridization System. After 16 h of hybridization, non-specific binding targets were washed away. The slides were scanned using a DNA Microarray Scanner (Model G2505C, Agilent Technologies, Santa Clara, CA, USA). The intensity of Cy5 fluorescence for each spot was analyzed by GenePix 4.1 software (Molecular Devices, Sunnyvale, CA, USA). Each single sample was analyzed at least twice in terms of technical or biological replicates, with a resulting reproducibility of r > 0.975 (Pearson's correlation coefficient). The signal intensity data were loaded into the Rosetta Resolver System (Rosetta Biosoftware, Seattle, WA, USA) for data preprocessing, which included centering normalization at the 75th percentile. The errors of the sample were estimated by using the error-weighted approach simultaneously. Both the fold change and P-value for pair-wise sample comparison were calculated to evaluate differentially expressed genes (DEGs). Rosetta Resolver 7.2 was employed to calculate fold changes, and the error model adjusted by Amersham Pairwise Ration Builder for signal comparison of sample. The statistical analysis was corrected with Empirical Bayes Statistics 12. The criteria for selection of significant DEGs were a FC ≥ 2 or ≤2 and P < 0.05. The microarray data were deposited in the National Center for Biotechnology Information Gene Expression Omnibus database (http://www.ncbi.nlm.nih.gov/gds), accession number GSE214910. (https://www.ncbi.nlm.nih.gov/geo/query/acc.cgi?acc= GSE214910).

### KEGG pathway and GO enrichment analysis of DEGs

Datasets representing genes with altered expression profiles derived from microarray analyses were generated using the “clusterProfiler” package in R software 13, 14*.* Gene Ontology (GO) and Kyoto Encyclopedia of Genes and Genomes (KEGG) were used to perform the pathway enrichment analysis for the DEGs. By calculating the similarity of their global annotation profiles with an agglomeration algorithm method, we classified the list of notable genes into clusters grouped under the headings 'biological process (BP),' 'cellular component (CC)', and 'molecular function (MF)'. As for both GO and KEGG pathway enrichment analysis, the cut-off criteria were P < 0.05 and P. adjust < 0.5. The Benjamini-Hochberg method was used to calculate the P. adjust.

**Ingenuity pathway analysis (IPA)**

Datasets representing genes with altered expression profiles derived from microarray analyses were uploaded into the Ingenuity Pathway Analysis (IPA) Tool (Ingenuity Systems, Redwood City, CA, USA; http://www.ingenuity.com) 15. IPA leverages the Ingenuity Pathway Knowledge Base (IPKB), sourced from previously published information on the known functions and interactions of genes. Thus, the IPA tool allows the identification of biological pathway and functional networks of a particular dataset. The IPA tool shows interactions between gene products and how these gene products act on each other (directly or indirectly). This includes genes that are not part of the microarray analysis.

### Statistical analysis

Data are presented as the mean ± standard deviation from three at least three independent experiments. Statistical analysis was performed using the Mann-Whitney test for two groups, while Kruskal-Wallis test followed by Dunn's multiple comparison test for the experimental groups against a single control group. Data analysis and graph generation were performed using GraphPad Prism 7 (GraphPad Software, Inc., La Jolla, CA, USA). P<0.05 was considered to indicate a statistically significant difference.

## Results

### HOXD10 affects EOC cell viability and motility

**Figure 1:**
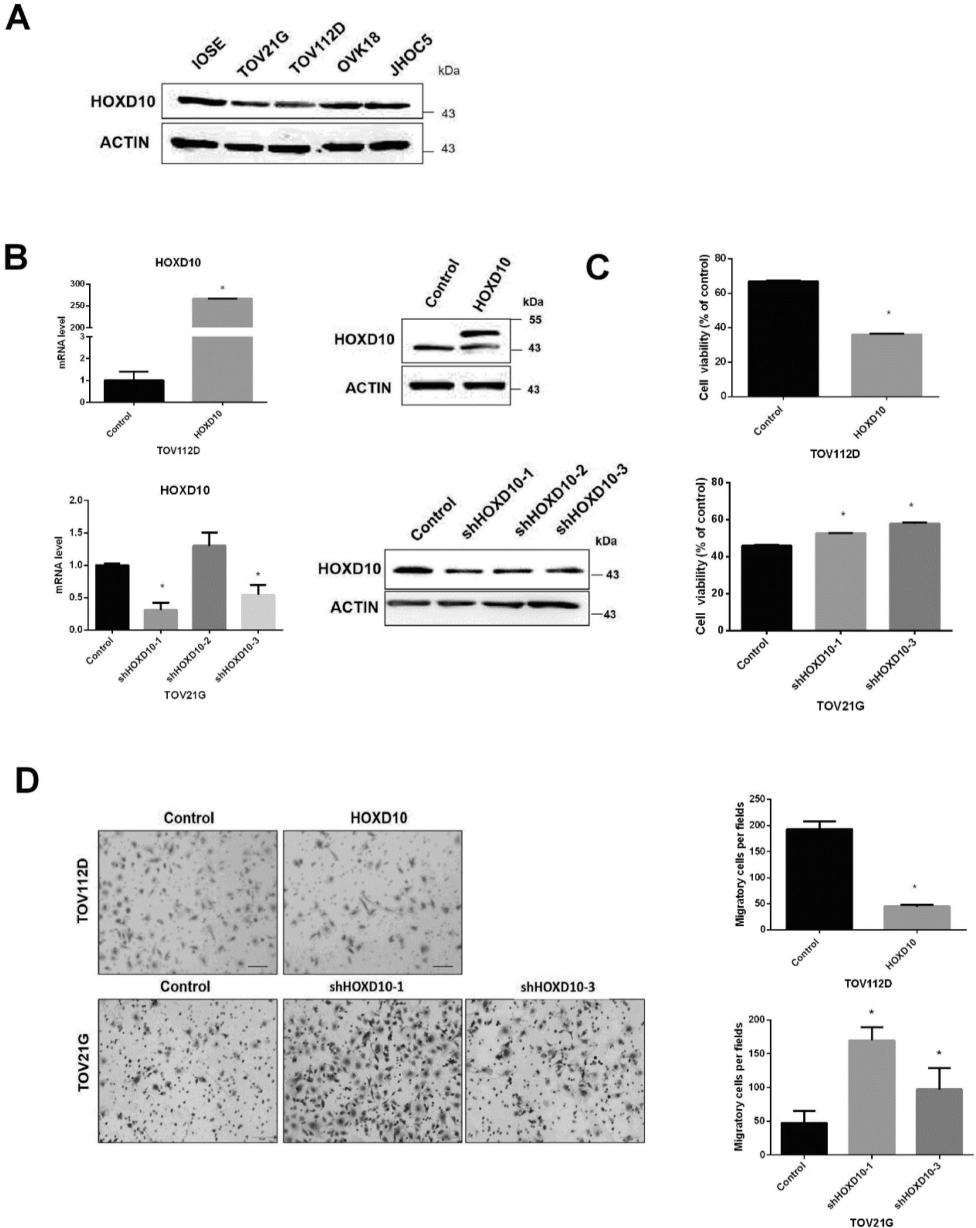
HOXD10 expression in ovarian cell lines. (A) Protein levels of HOXD10 in different ovarian cell lines. IOSE, immortalized normal human ovarian surface epithelial cells. TOV21G, TOV112D, JHOC5, and OVK18 are EOC cell lines. (B) qPCR and western blot analysis of HOXD10 following transfection of TOV112D cells with HOXD10 plasmid or control plasmid and TOV21G with HOXD10-shRNA or control shRNA. (C) A cell viability assay was performed onTOV112D cells transfected with HOXD10 plasmid and on TOV21G cells transfected with HOXD10-shRNAs. (D) A Transwell migration assay was performed on TOV112D cells transfected with HOXD10 plasmid and TOV21G cells transfected with HOXD10-shRNA. Representative images of the Transwell membranes are shown. Magnification, 100×. Data are presented as the mean ± standard deviation from three independent experiments. *P < 0.05.

We previously found that HOXD10 drives the expression of a microRNA (miRNA) associated with endometrioid ovarian cancer and clear cell ovarian cancer. To further investigate the potential role of HOXD10 in EOC, we analyzed its expression in IOSE and other human EOC cell lines, including TOV21G, TOV112D, JHOC5, and OVK18, via western blotting. IOSE cells are typically used as being representative of normal ovarian tissue. The levels of HOXD10 in EOC cell lines were notably decreased as compared with those in IOSE (Fig. 1A). HOXD10 has been implicated in regulating cell migration and invasion in several types of cancer cells 16, 17. Thus, we examined the role of HOXD10 in the regulation of ovarian cancer cell proliferation, and migration by introducing HOXD10 plasmid or HOXD10-shRNA. First, we confirmed that HOXD10 mRNA and protein expression were efficiently increased compare with control by transfection with HOXD10 plasmid in TOV112D cells and were decreased by transfection with HOXD10-shRNA in TOV21G cells (Fig. 1B). The cell viability and migration abilities of TOV112D cells were remarkably reduced after introducing HOXD10, whereas inhibiting HOXD10 increased cell viability and migration in TOV21G cells (Fig. 1C and D). These results indicated that HOXD10 could affect the proliferative capacity and motility of ovarian cancer cells.

### Identification of DEGs in EOC cells that overexpress HOXD10

**Figure 2:**
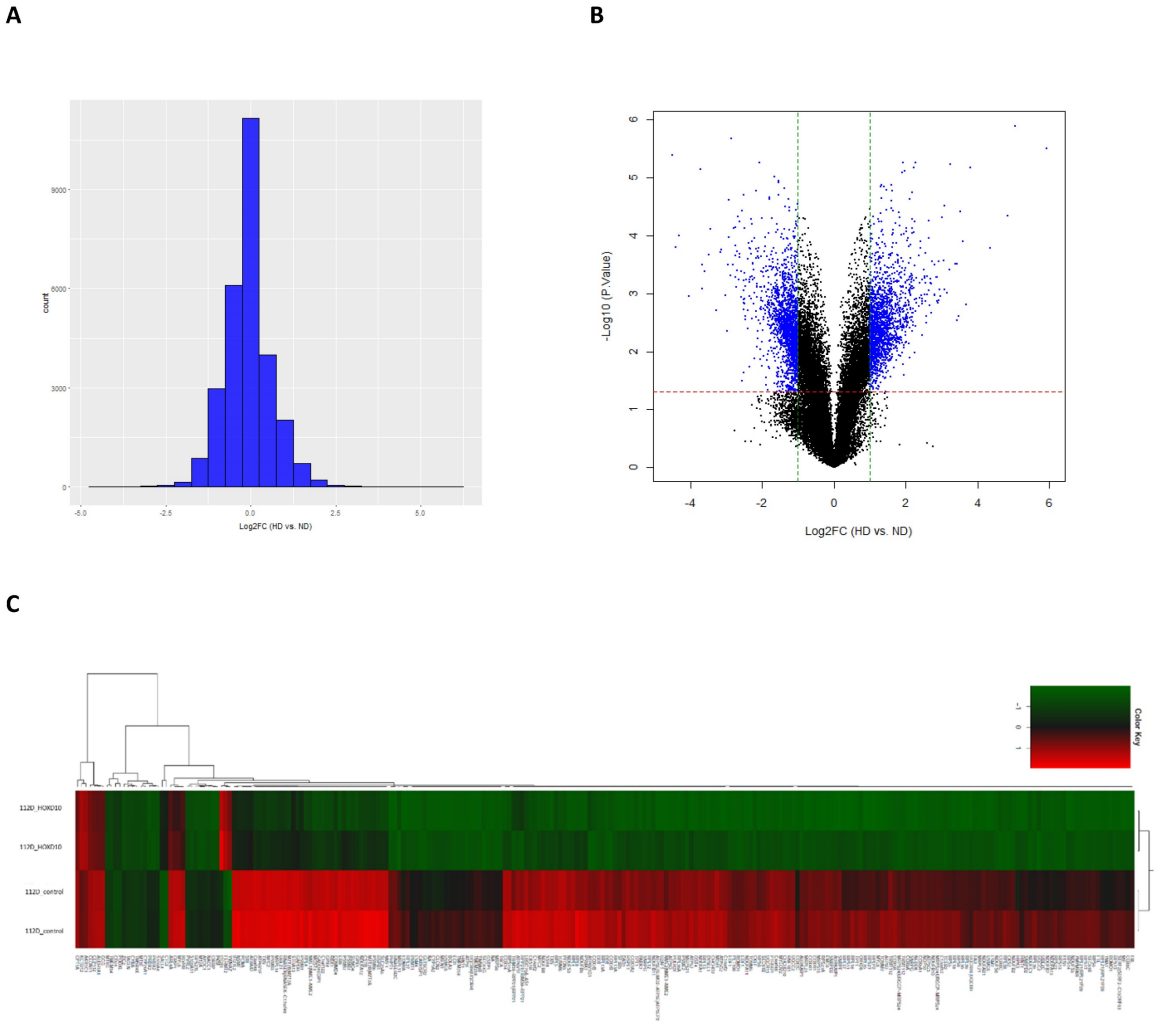
Identification of differentially expressed genes (DEGs). (A) The histogram shows the distribution of the gene expression ratios between HD and control cells (HD vs ND). The horizontal axis indicates the expression ratio, and the vertical axis indicates number of genes. (B) Volcano plot of HD vs. ND expression data. The red and green dashed lines represent the cut-off values for DEGs. DEGs are shown as blue dots. (C) Heatmap and hierarchical cluster analysis of functionally characterized DEGs for HD and ND cells. Red indicates the upregulated genes and green indicates the downregulated genes. HD, HOXD10 transfection; ND, control plasmid.

To investigate the underlying mechanism responsible for the effects of HOXD10 in EOC, we overexpressed HOXD10 in epithelial ovarian cells lines, and cells were collected for total RNA extraction for gene microarray analysis (Fig. S1). The histogram and volcano plot show the distribution of gene expression ratios between the HOXD10 transfection (HD) group and the control (ND) cells (Fig. 2A and 2B). Standard selection criteria used to identify DEGs were used based on the FC at |log_2_FC| ≥ 1 and P < 0.05 (log_2_FC ≥ 1.0 means upregulated and log2Fc ≤ -1.0 means downregulated) (Fig. 2B). Based on the cut-off criteria HOXD10 overexpression was associate with 1,508 upregulated genes and 1,560 downregulated genes (Table S1). Heatmap and hierarchical clustering analysis showed that HOXD10 over-expression affected gene clusters in EOC (Fig. 2C). Expression profiles were well correlated between the HD and ND groups as demonstrated by an unsupervised hierarchical clustering analysis tree. The top 10 upregulated and downregulated DEGs in the presence of HOXD10 overexpression are shown in Table 1.

### Functional enrichment analysis of DEGs

**Figure 3:**
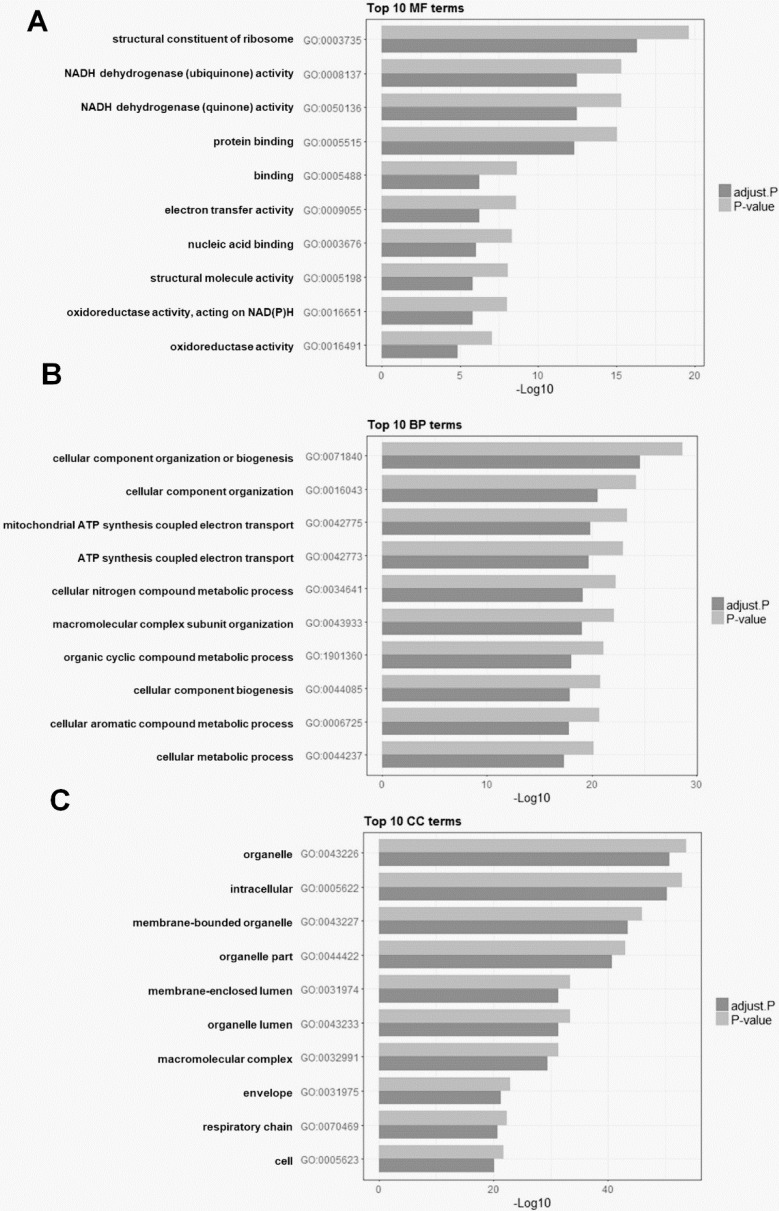
The top 10 enriched Gene Ontology (GO) analysis terms for DEGs after HOXD10 overexpression. (A-C) Bar charts showing the top 10 GO terms for (A) molecular function (MF), (B) biological process (BP), and (C) cellular component (CC). P-values were adjusted with the false discovery rate calculated using the Benjamini-Hochberg procedure.

To explore the biological significance of HOXD10 in EOC, a KEGG pathway and GO analyses were used for the functional annotation of DEGs. A total of 3,068 GO terms were significantly enriched. The DEGs were categorized into three functional groups: molecular function, cellular component, and biological process. The top 10 enriched GO terms in each functional group are shown for the isolate DEGs. DEGs were significantly enriched for the terms of NADH dehydrogenase activity and ATP synthesis coupled electron transport, for example (Fig. 3). To investigate pathways related to these DEGs, gene expression information was mapped to the KEGG pathways. As shown in Table 2, the enriched pathways were identified for KEGG pathway analysis. KEGG pathway analysis showed that DEGs were enriched for the terms of oxidative phosphorylation, ribosome, and pyrimidine metabolism.

**Figure 4:**
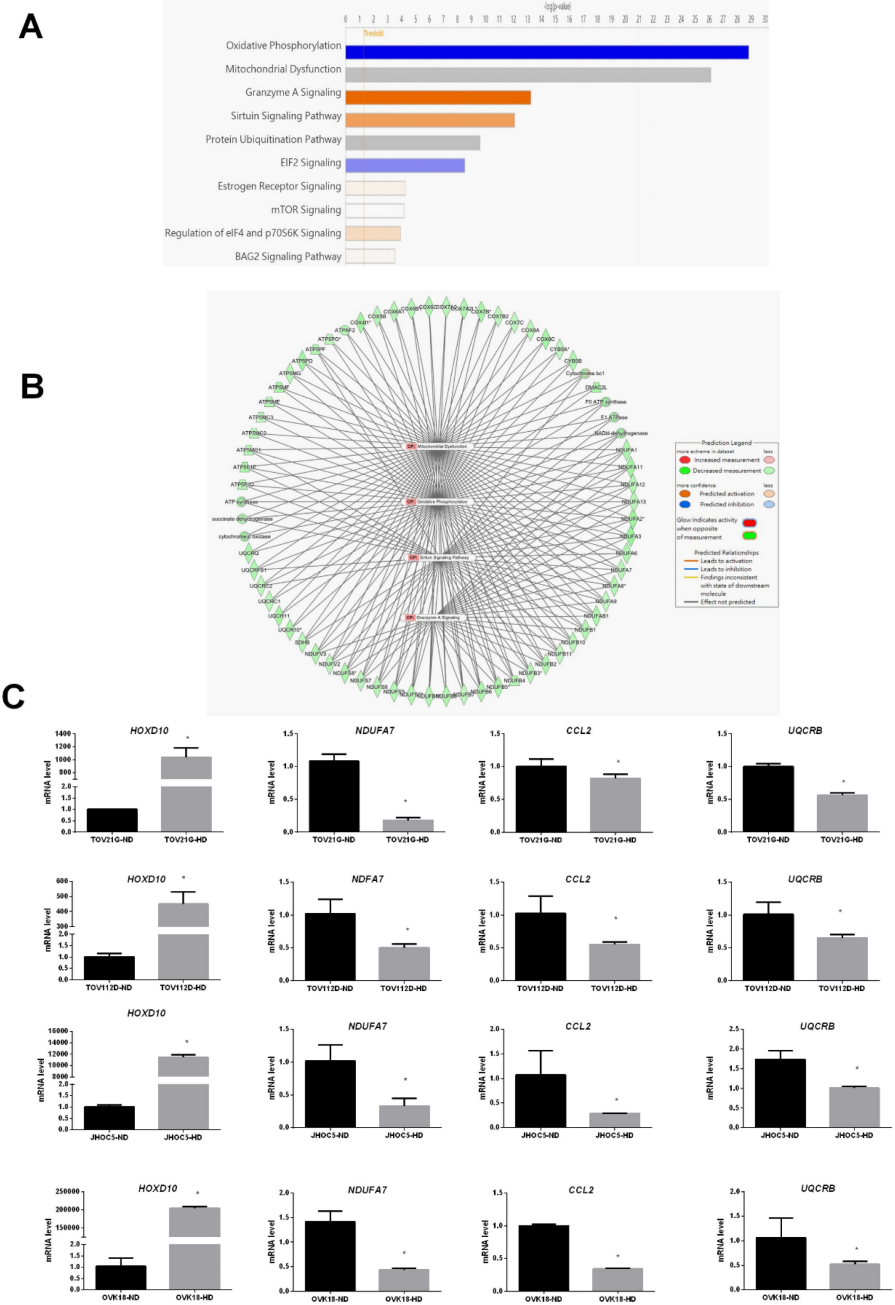
Canonical pathway analysis of DEGs after overexpression of HOXD10 in EOC cells. (A) The top 10 canonical pathways derived from the ingenuity pathway analysis (IPA). (B) The visual network of pathway interactions among genes. The relevant network among oxidative phosphorylation, mitochondria dysfunction, granzyme A signaling pathway genes is shown, and sirtuin signaling pathway. (C) qPCR analysis of *HOXD10*, *NDUFA7, UQCRB* and* CCL2* after transfection of TOV21G, TOV112D, JHOC5 and OVK18 cells with HOXD10 plasmid. Expression is shown relative to the control *P < 0.05. HD, HOXD10 transfection; ND, control plasmid. Data are presented as the mean ± SD. Blue bars: negative z-score; orange bars: positive z-score; gray bars: no activity pattern available.

We also used IPA to identify the most significantly enriched canonical pathways and examine the diseases and biofunctions associated with these DEGs. The DEGs were enriched in different pathways, among which oxidative phosphorylation, mitochondrial dysfunction, granzyme A signaling, the sirtuin signaling pathway, and the protein ubiquitination pathway were the most significantly affected (Fig. 4A). All categories of canonical pathways and their associated genes are listed in Table S2. to the category that was most enriched was oxidative phosphorylation (Table 2 and Fig. 4A). The HOXD10-regulated network of potential target gene is shown in Figure 4B. Potential interactions among the differentially expressed pathways are depicted in this network and include genes related to oxidative phosphorylation, mitochondria dysfunction, the granzyme A signaling pathway, and the sirtuin signaling pathway.

To confirm the reliability of the fold changes identified by the microarray analysis, we further evaluated the expression of *NDUFA7*, *UQCRB* and *CCL2* in the presence of HOXD10 overexpression in TOV112D, TOV21G, JHOC5 and OVK18 cells. *NDUFA7, UQCRB* and *CCL2* expression was significantly changed by HOXD10 overexpression (Fig. 4C). Those *NDUFA7*and *UQCRB* are among those signaling pathway and involved in OXPHOS complex subunits. NDUFA7 is an important component of mitochondrial complex I, and UQCRB is a comprised subunit of mitochondrial complex III. CCL2 is one of the top 10 DEGs.

In addition to canonical pathways, DEGs were categorized in terms of related diseases and functions. Table 3 shows the top five significantly enriched items in the three categories. All categories of diseases and functions and their associated genes are listed in Table S3. These significantly enriched terms should help us to further understand the roles of that these DEGs in EOC.

## Discussion

Our previous study showed that overexpression of HOXD10 reverses the low expression of miR-381 in endometriosis-associated clear cell and endometrioid ovarian cancer 9. Given that HOXD10 drives the expression of miR‑381, it may affect the proliferation and metastasis of EOC. HOXD10, a tumor suppressor gene that is expressed at a low level in most tumors compared with normal tissues. 18. An IOSE cells are typically used as being representative of normal ovarian tissue. In our data showed that HOXD10 is lowly expressed in EOC cells compared with IOSE, which is consistent with previous studies in tumor. Exploring the regulatory pathways and downstream genes associated with HOXD10 provide new biological indicators for the early detection of EOC or novel avenues for treatments of ovarian cancer.

In the current study, microarray and bioinformatics analysis were used to examine the DEGs in EOC cells after HOXD10-overexpression. A total of 3,068 DEGs were identified, including 1,508 upregulated and 1,560 downregulated DEGs (Table S1). Moreover, Bioinformatics enrichment tools were used for pathway and functional analyses. In this study, KEGG pathway enrichment analysis demonstrated that one of the top pathways was oxidative phosphorylation (Table 2). IPA showed that the top significantly changed canonical pathways were related to oxidative phosphorylation, mitochondrial dysfunction, granzyme A signaling, sirtuin signaling pathway, and protein ubiquitination pathway (Fig. 4A). GO enrichment analysis and IPA identified that the most important function of the identified DEGs in HOXD10 overexpression was associated with oxidative phosphorylation and mitochondrial function (Fig. 3A, B and Fig. 4A). On the basis of microarray and bioinformatic analyses, oxidative phosphorylation and mitochondrial function were affected by HOXD10 expression in these cells, which could lead to provide the potential therapeutic strategies to target EOC. The oxidative phosphorylation systems (OXPHOS) are responsible for producing ATP molecules in mitochondria, which participate in the generation and regulation of cellular bioenergetics. The Warburg effect and concomitant metabolic alterations in cancers have led to a consensus that mitochondrial dysfunction occurs 19. Dysfunction oxidative phosphorylation occurs frequently in many tumors. Dysfunction oxidative phosphorylation metabolic pathway occurs frequently in many tumors, including ovarian cancer tumors 20, 21. In addition, the level of *NDUFA7* and *UQCRB*, which are components of mitochondrial complex I and III, respectively, were significantly changed by HOXD10 overexpression (Fig. 4C). A recent study revealed that CCL2 affected mitochondrial function, including oxidative phosphorylation in tissue, suggesting CCL2 link to metabolism 22. These findings suggested that HOXD10 may regulate the progression to EOC by affecting OXPHOS. Growing evidence shows that metabolic adaptation is an emerging hallmark of tumors 23. Thus, the findings of the present study are consistent with those of previous studies. Cancer cells usually show adaptations in their metabolism for the regulation of gene and protein expression and to influence the behavior of non-transformed cells in the vicinity of the tumor. This facilitates the growth, invasiveness, and metastasis of these cells and is associated with resistance to therapies. In addition, the GO of the DEGs in this study indicated enrichment for biological processes, such as granzyme A signaling and sirtuin signaling, which are associated with energy metabolism and tumorigenesis 24, 25. Despite there being many chemotherapeutic agents and treatment protocols available for ovarian cancer, cancer drug resistance, which is one of the leading causes for cancer therapy failure and recurrence, has been a major problem for treating this cancer. A consequence of metabolic adaptation to the tumor microenvironment known as metabolic reprogramming, is a distinctive characteristic of chemoresistant cancer cells. In addition to oxidative phosphorylation, mitochondrial dysfunction was also implicated in the metabolic reprogramming of these EOC cells and is involved in several chemoresistance pathways 26. HOXD10 therefore has an important role in metabolism-related pathway with EOC. Thus, the data suggest that strategies to interfere with metabolism-related pathways associated with cancer drug resistance could be considered for the treatment of ovarian tumors.

Oxidative metabolism drives inflammation-induced platinum resistance in human ovarian cancer 27. In the present study, canonical pathway enrichment identified granzyme A signaling, which is implicated in multiple physiological processes including cellular metabolism, apoptosis and inflammatory responses 28, 29. Granzyme A signaling can act as a pro-inflammatory mediator and induce pro-inflammatory cytokines in several cell types including monocytes, macrophages, endothelial and epithelial cells and fibroblasts, and then affects tumor microenvironments 29. Thus, Granzyme A signaling is not only associated with energy metabolism but also acts as an inflammatory mediator. HOXD10 was reported to be associated with the epithelial-mesenchymal transition (EMT), which plays a central role in cancer progression, metastasis and drug resistance, and induced by inflammation. Thus, HOXD10 may be involved in cancer development through the alteration of the tumor microenvironment by granzyme A.

In conclusion, in the present study, we analyze the potential mechanism underlying HOXD10 through microarray data of EOC cells. Our results showed that HOXD10 is involved in cancer metabolism and tumor microenvironment in ovarian cancer. Cancer metabolism and tumor microenvironment are linked to chemotherapeutic resistance, which is currently one of the most important challenges in cancer treatment. Thus, the data suggesting that HOXD10 is a potential target for treating EOC. Our results therefore provide new insights into drug resistance mechanism in EOC that may lead to the design of novel therapeutic approaches. In addition, the variation in gene expression related to oxidative phosphorylation and mitochondrial function may lead to the development of prognostic or therapeutic biomarker for EOC.

The present study has a few limitations. First, the study is lack of clinical tissue validation. The validation in clinical tissue samples will be necessary to confirm the DEGs in the further study. It expected that these DEGs may serve as potential targets for therapy or prognosis biomarker. In addition, RNA-seq and microarray platform are two major approaches for conducting transcriptional profiling. RNA-Seq has emerged as an alternative to microarray platforms for analyzing DEGs, since it is more sensitive than microarray, particularly among genes expressed at low levels. Compared to RNA-seq, microarray data is manageable in size and well-established in data processing and analyses.

The overall computation time are much lower in microarray experiment. In our study, we use the Human OneArray microarray which is the one of the most powerful tools for analysis of gene or protein expression. The main limitation of microarrays is inability to detect genes with low expression levels and only can detect those sequence for which they designed. Several of studies comparing the RNA-Seq and microarray differential expression detection showed a high correlation between gene expression profiles generated by these two platforms 30-32. Since each method has its own disadvantage, they can be complemented with each other.

## Supplementary Material

Supplementary figure and tables.

## Figures and Tables

**Table 1:** The top 10 DEGs associated with HOXD10 overexpression in TOV112D

Gene symbol	Description	Fold change
**Upregulated with HOXD10 overexpression**		
OTUD4	OTU deubiquitinase 4	5.920443469
GPNMB	Glycoprotein	4.837110459
AMIGO2	Adhesion molecule with Ig-like domain 2	4.357560328
CSAG1	Chondrosarcoma associated gene 1	3.807386027
GJA3	Gap junction protein, alpha 3, 46kDa	3.691999894
JAG1	Jagged 1	3.588596006
GREM1	Gremlin 1, DAN family BMP antagonist	3.523690754
HAUS3	HAUS augmin-like complex, subunit 3	3.468420095
ADGB	Androglobin	3.436879326
TMEM100	Transmembrane protein 100	3.383809323
**Downregulated with HOXD10 overexpression**		
SERTAD4-AS1	SERTAD4 antisense RNA 1	-4.405973321
LOX	Lysyl oxidase	-4.327442941
CDH12	Cadherin 12, type 2	-4.038917551
PRDX2	Peroxiredoxin 2	-3.722692908
MEIS1	Meis homeobox 1	-3.681582135
CXCL14	Chemokine	-3.662821577
IGFBP6	Insulin-like growth factor binding protein 6	-3.592634447
ZDHHC24	Zinc finger, DHHC-type containing 24	-3.439141408
CCL2	Homo sapiens chemokine (C-C motif)	-3.165796477
HDAC9	Homo sapiens histone deacetylase 9 (HDAC9)	-3.149266703

**Table 2:** Pathways from the Kyoto Encyclopedia of Genes and Genomes (KEGG) enrichment analysis

Description	P-value	P.adjust^†^	Count
Oxidative phosphorylation	1.32E-21	1.45E-19	67
Ribosome	2.04E-09	8.94E-08	38
Pyrimidine metabolism	8.33E-04	2.32E-02	28
Spliceosome	8.49E-04	2.32E-02	34
Pathways in cancer	3.73E-03	9.08E-02	69
Proteasome	6.54E-03	1.43E-01	14
TGF-beta signaling pathway	9.15E-03	1.72E-01	22
RNA polymerase	9.43E-03	1.72E-01	10
NOD-like receptor signaling pathway	1.56E-02	2.29E-01	16
RNA transport	1.57E-02	2.29E-01	34
RNA degradation	2.14E-02	2.93E-01	18
Toxoplasmosis	3.34E-02	4.16E-01	29

^†^ P-values were adjusted with the false discovery rate calculated using the Benjamini- Hochberg procedure.

**Table 3:** Top five Bio Function categories altered after HOXD10 overexpression

Name	P-value range	No. of molecules
**Diseases and Disorders**		
**Cancer**	9.90E-03-2.93E-11	1871
**Organismal Injury and Abnormalities**	9.90E-03-2.93E-11	2180
**Reproductive System Disease**	8.69E-03-2.93E-11	716
**Gastrointestinal Disease**	9.90E-03-2.93E-11	1247
**Developmental Disorder**	9.16E-03-6.86E-11	601
		
**Molecular and Cellular Functions**		
**Cell Signaling**	6.06E-03-1.76E-11	105
**Post-Translational Modification**	9.82E-03-1.76E-11	280
**Protein Synthesis**	8.10E-03 -1.76E-11	272
**Cell Death and Survival**	9.05E-03-2.91E-11	633
**Gene Expression**	3.79E-03-6.51E-08	392
		
**Physiological System Development and Function**		
**Tissue Development**	8.68E-03-5.36E-05	79
**Renal and Urological System Development and Function**	6.63E-03-1.20E-04	76
**Hair and Skin Development and Function**	8.30E-03-2.34E-04	96
**Organismal Development**	9.90E-03-3.07E-04	58
**Embryonic Development**	9.90E-03 -6.25E-04	129

The categories were identified using the Ingenuity Pathway Analysis (IPA) software.
