# Supplementary figures and images for: Gene Expression Profile Analysis of the Molecular Mechanism of HOXD10 Regulation of Epithelial Ovarian Cancer Cells
Source: J Cancer. 2024 Jan 1;15(5):1213–24. doi: 10.7150/jca.90970 (PMC10861814; doi:10.7150/jca.90970)

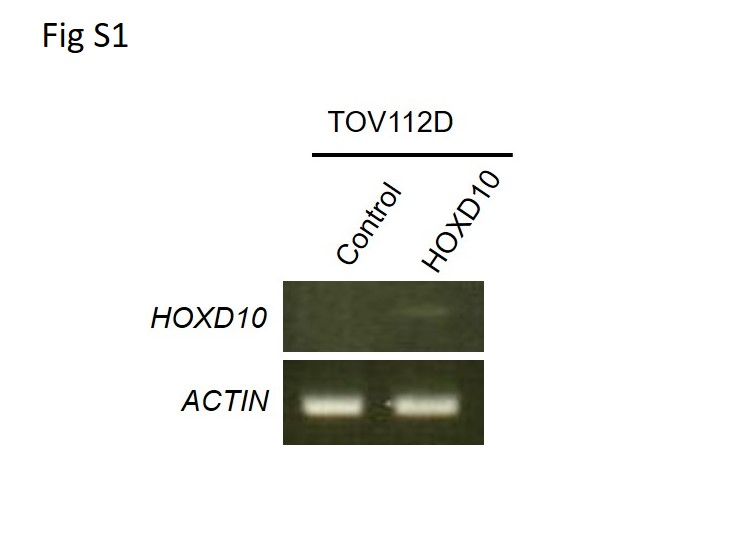

Supplement: Supplementary file 1 — Supplementary figure and tables. [file jcav15p1213s1.zip › supplementary/Fig S1-.tif]
